# A Retrospective Comparative Study of Modified Percutaneous Endoscopic Transforaminal Discectomy and Open Lumbar Discectomy for Gluteal Pain Caused by Lumbar Disc Herniation

**DOI:** 10.3389/fsurg.2022.930036

**Published:** 2022-06-22

**Authors:** Junyan An, Jun Zhang, Tong Yu, Jiuping Wu, Xinyu Nie, Tao He, Zhihe Yun, Rui Liu, Wu Xue, Le Qi, Yingzhi Li, Qinyi Liu

**Affiliations:** Department of Orthopedics, The Second Hospital of Jilin University, Changchun, China

**Keywords:** lumbar disc herniation, gluteal pain, percutaneous endoscopic transforaminal discectomy, open lumbar discectomy, minimally invasive surgery

## Abstract

**Introduction:**

This study aimed to demonstrate the safety and effectiveness of modified percutaneous endoscopic transforaminal discectomy (PETD) in the surgical management of single-segment lumbar disc herniation (LDH) gluteal pain and to determine whether it provides a better clinical outcome than open lumbar discectomy (OD).

**Methods:**

A retrospective analysis of patients treated with modified PETD and OD for gluteal pain in LDH from January 2015 to December 2020 was conducted. Sample size was determined using a priori power analysis. Demographic information, surgical outcomes including procedure time (minutes), intraoperative blood loss (mL), hospital days, costs (RMB), fluoroscopy shots, recurrence and complications, etc., were recorded and analyzed. Prognostic outcomes were assessed using the visual analog scale (VAS), the Oswestry Disability Index (ODI), the Japanese Orthopedic Association Score (JOA) and modified MacNab criteria. The preoperative and postoperative VAS, ODI and JOA scores were recorded by two assistants. When the results were inconsistent, the scores were recorded again by the lead professor until all scores were consistently recorded in the data. MRI was used to assess radiological improvement and all patients received follow-ups for at least one year.

**Results:**

The sample size required for the study was calculated by a priori analysis, and a total of 72 participants were required for the study to achieve 95% statistical test power. A total of 93 patients were included, 47 of whom underwent modified PETD, and 46 of whom underwent OD. In the modified PETD intragroup comparison, VAS scores ranged from 7.14 ± 0.89 preoperatively to 2.00 ± 0.58, 2.68 ± 0.70, 2.55 ± 0.69, 2.23 ± 0.81, and 1.85 ± 0.72 at 7 days, 1 month, 3 months, 6 months, and 12 months postoperatively. Patients showed significant pain relief postoperatively (*P* < 0.01). According to the modified MacNab score, the excellent rate in the PETD group was 89.36%. There was no significant difference compared to the OD group (89.13%, *P* > 0.05). Complication rates were lower (*P* > 0.05) but recurrence rates were higher (*P* > 0.05) in the modified PETD group than in the OD group. The modified PETD group had a faster operative time (*P* < 0.01), shorter hospital stay (*P* < 0.01), less intraoperative bleeding (*P* < 0.01), and less financial burden to the patient (*P* < 0.01) than the OD group. At 7 days postoperatively, the VAS score for low back pain was higher in the OD group than in the modified PETD group (*P* < 0.01). The VAS and JOA scores at 1, 3, 6, and 12 months postoperatively were not significantly different between the modified PETD and OD groups (*P* > 0.05), and the ODI was significantly different at 3 months postoperatively (*P* < 0.05).

**Conclusion:**

Modified PETD treatment is safe and effective for gluteal pain due to L4/5 disc herniation and has the advantages of a lower complication rate, faster postoperative recovery, shorter length of stay, fewer anesthesia risks and lower cost of the procedure compared with OD. However, modified PETD has a higher recurrence rate.

## Introduction

Lumbar disc herniation (LDH) is one of the most common degenerative diseases of the lumbar spine, typically causing lower back pain and sciatica (1, 2). Gluteal pain has often been a clinical manifestation, and sometimes the only manifestation, of patients with LDH (3).

In a retrospective study reported by Fang et al. (3), 94.64% of patients with gluteal pain had responsible L4/5 segments (*P* < 0.001), and 5.36% had L5/S1. Wang et al. (4) subsequently described the mechanism of gluteal pain in LDH and suggested that it may be related to the superior and inferior gluteal nerves. All the fibers of the anterior branch of the L5 nerve root form the lumbosacral trunk, which forms part of the sacral plexus and branches distally into the superior gluteal nerve (L4, L5, S1) and the inferior gluteal nerve (L5, S1, S2), innervating the sensory muscles of the gluteal region, respectively (5, 6). In addition, compression of the posterior branch of the spinal nerve may contribute to gluteal pain, as the anterior and posterior roots merge at the intervertebral foramen to form the spinal nerve, which immediately divides into the posterior branch, creating a thicker nerve trunk that includes the superior cluneal nerves. Previous studies (7, 8) have shown that in addition to L1, L2, and L3, the posterior branches of the L4 and L5 spinal nerves are also involved in the formation of the superior cluneal nerves. Further autopsies have confirmed that approximately 10% of the superior cluneal nerves originate from L5 (9), a group of purely sensory nerve fibers controlling the gluteal region (10, 11). This reveals a strong correlation between gluteal pain and L4/5 disc herniation.

In terms of surgical treatment, OD remains the standard of care for pain secondary to LDH (12, 13), which is performed via a posterior approach, where the epidural space is exposed in the posterior midline by separating the paravertebral muscles as well as excising the lamina and ligamentum flavum. The herniated disc is removed after excision of a section of the facet joint on the symptomatic side while protecting the spinal cord and nerve roots (14). Although OD is effective, it can also cause considerable tissue damage (15).

With the development of minimally invasive methods, PETD is rapidly replacing OD in procedures requiring discectomy and decompression (16). Experienced surgeons can reach the lesion directly through Kambin’s triangle bypass (17). PETD avoids extensive damage to the skin, muscles, laminae, and synapses (18), and more significantly, excessive strain on the dural sac is avoided (19). Li et al. (14) also demonstrated that PETD achieved satisfactory results in the treatment of LDH with a reduced incidence of iatrogenic injury and minimal activity restrictions compared to OD, thus accelerating rapid recovery.

However, PETD focuses on the surgical approach and removal of the nucleus pulposus. The annulus fibrous and posterior longitudinal ligament, which may cause gluteal pain, are not treated or described in detail (20). Therefore, this study investigated a modified PETD that hypothesized that resection of the annulus fibrosus and posterior longitudinal ligament might significantly reduce pain in patients. The purpose of this study was to assess the safety and efficacy of a modified PETD compared with OD for treating L4/5 single-segment disc herniation.

## Materials and Methods

### Patients

The clinical study was approved by the Chinese Ethics Committee (No. 2021001). We recruited patients who underwent either the modified PETD technique or OD patients for LDH at our institution from January 2015 to November 2020 and were followed up for at least one year. Telephone follow-ups were carried out at each follow-up time, and basic information about all patients was reviewed. In addition, patients were invited to undergo reexamination to observe their most recent clinical and radiological results.

**Figure 1:**
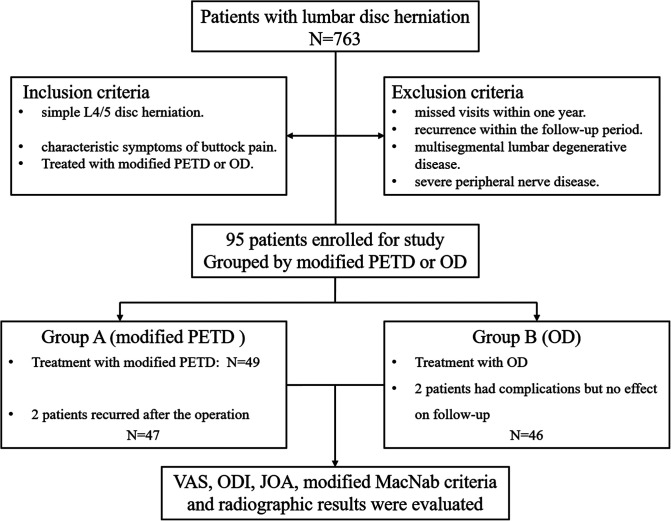
Flow chart of patient inclusion and stratification. Modified PETD, modified percutaneous endoscopic transforaminal discectomy; OD, open lumbar discectomy.

The inclusion criteria were adult patients with single-segment L4/5 disc herniation with only symptoms of gluteal pain. Patients chose to be treated with either a modified PETD technique or OD. The exclusion criteria were as follows: a previous history of lumbar operation; missed visits within one year or recurrence within the follow-up period; multisegment lumbar degenerative disease; and severe peripheral nerve disease (Figure 1). Recurrence was defined as a recurrence of the same level of disc herniation, and reoperation was performed.

### Preoperative

All patients underwent preoperative magnetic resonance imaging (MRI) of the lumbar spine, computed tomography (CT), and lumbar X-ray plain radiographs (anterior and lateral views). The same physician treated all patients. The modified PETD technique was performed using local anesthesia, and patients were informed of the potential for intraoperative discomfort and pain. A transilluminated surgical bed and C-arm were used for intraoperative positioning. Normal saline (3,000 mL) was used for continuous irrigation via the endoscope.

### Operative

The routine procedure was as described in a previous study (21). In brief, the patient was operated on in a lateral position with the affected side facing upward and a soft cushion on the lumbar area. The skin entry point was above the iliac crest, 12–14 cm from the midline. After local anesthesia, the superior articular eminence of the external L5 was fixed under C-arm guidance and infiltrated locally with additional anesthetic. A guidewire was inserted through an 18-gauge needle, and an incision of approximately 0.7 cm was made at the edge of the guidewire. A stepwise dilating catheter was placed along the guidewire to bluntly separate the surrounding muscle tissue, place a working channel and connect to the endoscopic system. Physiological saline was continuously irrigated to ensure a clear view, and the protruding nucleus pulposus was removed using endoscopic forceps.

### Denervation of the Annulus Fibrous

**Figure 2:**
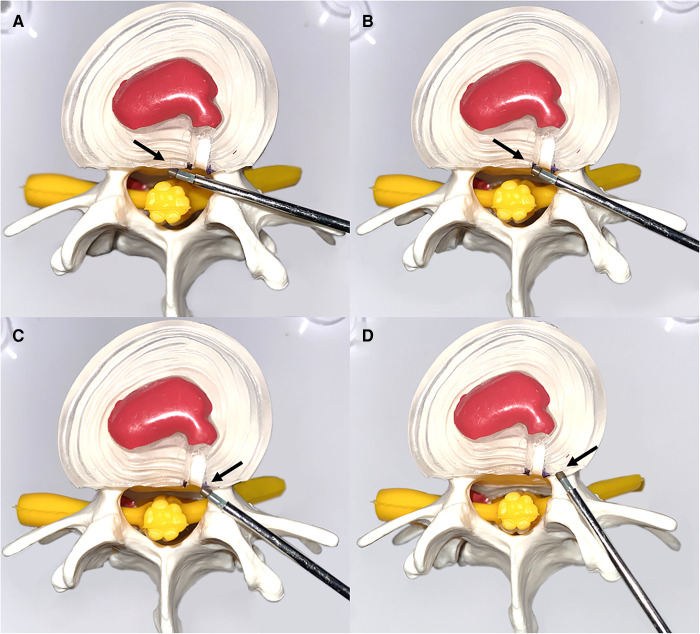
Schematic representation of the denervation of an annulus fibrosus. (**A**) Denervation from the posterior longitudinal ligament (black arrow). (**B,C**) Focused treatment of radiofrequency ablation of an annulus fibrosus dissection (black arrow). (**D**) Final denervation of the annulus fibrosus superior to the vertebral arch (black arrow).

After visualization of the symptomatic lateral annulus fibrous in endoscopic view, denervation of the annulus fibrosus was performed starting from the posterior longitudinal ligament at the posterior edge of the vertebral body up to the pediculus arcus vertebrae, with emphasis on radiofrequency ablation of the ruptured end of the annulus fibrosus. The proliferating nerves and vessels were eliminated (Figure 2).

### Excision of Hypertrophic Annulus Fibrous and Posterior Longitudinal Ligaments

**Figure 3:**
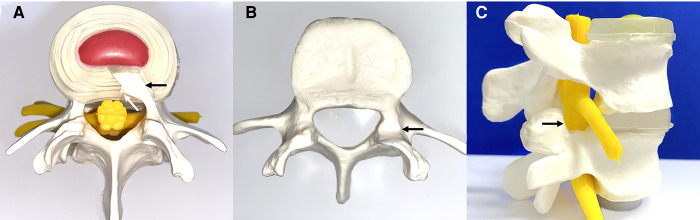
Schematic diagram of annulus fibrosus excision and lateral recess decompression. (**A**) Excision of the hypertrophic annulus fibrosus (black arrow) and posterior longitudinal ligament (white arrow). (**B,C**) Transverse and sagittal demonstration of lateral recess decompression with partial resection of the superior facet joint (black arrow).

After denervation, the hypertrophied annulus fibrous and posterior longitudinal ligament at the superior margin of the symptomatic inferior vertebral body was removed. The posterior longitudinal ligament was removed with endoscopic forceps (Figure 3A).

### Lateral Recess Decompression

**Figure 4:**
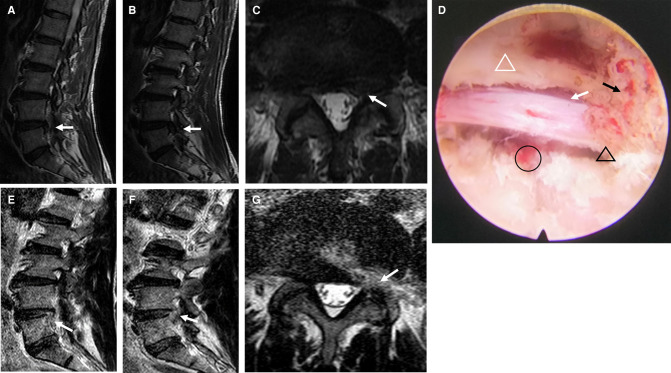
Pre- and postoperative images and intraoperative microscopic images of modified PETD. (**A–C**) Preoperative MRI demonstrated lumbar disc herniation (white arrows). (**D**) A completely decompressed nerve root is visible endoscopically (white arrow), with removal of the fibrous annulus (black circle), decompression of the lateral recess (black triangle), facet joint resection (black arrow), and partial ligamentum flavum resection (white triangle). After modified PETD, (**E,F**), the fibrous ring at the superior margin of the inferior conus was removed, and lateral saphenous fossa decompression was performed (**G**) (white arrow).

An endoscopic circular saw and osteotome were used to remove the hyperplastic superior facet joint up to the superior edge of the vertebral arch. A portion of the ligamentum flavum was removed to completely decompress the “peripheral recess” (Figure 3B). The endoscopic view showed good nerve root pulsation and complete decompression. Fluid gelatin was injected before removing the working cannula to prevent hematoma, and finally, the wound was sutured. A preoperative and postoperative MRI comparison showed complete removal of the nucleus pulposus and decompression of the lateral saphenous fossa (Figure 4).

### Clinical Assessment

Demographic information included age, sex, body mass index (BMI), smoking habit, alcohol consumption, hypertension, diabetes, duration of symptoms, side of symptoms, and follow-up time. Surgical outcomes included the duration of surgery, intraoperative blood loss, length of hospital stay, cost of surgery, number of radiation sessions, recurrence, and complications. Recurrence was defined as a reherniation of the disc at the same segment and on the same side with a VAS score >4. The prognostic outcome was assessed by the outcome values and improvement rates of VAS, ODI, JOA, and the modified MacNab criteria, where the primary outcomes are the outcome values and the improvement rates of VAS. The improvement rates for VAS and ODI were calculated using the (preoperative-postoperative)/preoperative formula for the results and the (postoperative-preoperative)/(29-preoperation) formula for JOA. The VAS is a subjective numerical pain scale that assesses the gluteal pain experienced by the patient in the last 24 h. The ODI, JOA and modified MacNab criteria are used to measure the degree of disability and treatment for the life of patients with gluteal pain, reflecting the recovery of function and the ability of patients to manage daily life after surgery (22).

### Statistical Analysis

We used G-POWER Analysis (Version 3.1.9.7) (23, 24) to obtain the minimum sample size required to achieve a medium effect (effect size, d = 0.25), a power of 95%, and a statistical significance level of 0.05. To achieve statistical significance, we found that at least 72 samples were required. IBM SPSS Statistics (version 23.0) was used for data analysis. The data are expressed as the mean ± standard deviation (SD) and frequency (percentage). The two groups were compared using Pearson chi-square tests or Fisher exact tests for categorical variables and independent samples t tests or Mann–Whitney tests for continuous variables. Outcome values and improvement rates for VAS, ODI, JOA and excellent rates for modified MacNab criteria were compared between groups using multivariate analysis. Modified PETD intragroup comparisons were performed using two-way repeated-measures ANOVA. *P* values <0.05 were considered statistically significant. All graphs were constructed with GraphPad Prism (version 8.0.2).

## Result

### Demographic Information and Surgical Outcomes

The results of the a priori power analysis indicated that the study required at least 72 subjects. A total of 93 participants eventually met the inclusion criteria, of whom 49 opted for the modified PETD technique and 46 patients for OD. All participants had unilateral gluteal pain, and the type of LDH was paramedian. The mean follow-up times were 15.98 ± 4.23 and 16.11 ± 4.32 months for the modified PETD and OD groups, respectively. Demographic information, including age, sex, BMI smoking, alcohol, hypertension, diabetes, duration of symptoms, side of symptoms and follow-up time, were not significantly different between the two groups (*P* > 0.05). Compared to the OD group, the modified PETD group had a significantly shorter operative time (*P* < 0.01), less intraoperative bleeding (*P* < 0.01), and a shorter hospital stay (*P* < 0.01). In addition, the modified PETD technique imposed a smaller financial burden on the patient (*P* < 0.01). However, there were fewer fluoroscopic shots in the OD group (*P* < 0.01) (Table 1).

**Table 1:** Demographic information and Surgical outcomes.

Characteristic	Modified PETD	OD	*P*-Value
Number (No.)	49	46	
Age (Yrs)	52.98 ± 11.52	52.98 ± 10.48	0.843
Gender (M:F)	23:26	21:25	0.900
BMI	24.93 ± 2.46	24.14 ± 3.32	0.190
Smoking (Y)	43%	41%	0.878
Alcohol (Y)	39%	41%	0.648
Hypertension (Y)	29%	28%	0.973
Diabetes (Y)	18%	17%	0.682
Duration of symptom (Mos.)	4.53 ± 1.54	4.48 ± 1.39	0.863
Side of symptoms (L:R)	23:26	22:24	0.350
Follow-up times (Mos.)	15.98 ± 4.23	16.11 ± 4.32	0.974
Duration of operation (min)	65.25 ± 8.37	127.72 ± 13.47	<0.01*
Blood loss (mL)	32.08 ± 4.79	126.26 ± 6.36	<0.01*
Hospital stays (day)	3.00 ± 0.35	7.11 ± 1.23	<0.01*
Costs (RMB)	3.55 [3.30, 3.80]	6.18 [5.78, 6.50]	<0.01*
Fluoroscopy shots	6.37 ± 0.86	3.59 ± 0.83	<0.01*
Recurrence	4%	0%	0.495
complications	0%	4%	0.232

*Patients are classified according to different surgical procedures. Data are presented as the mean ± standard deviation or number (%).*

*No., number; Yrs, years; M, male; F, female; Y, yes; Mos., months; L, left; R, right.*

*Significant difference between the two groups (P < *0.05)*.

### Prognostic Outcomes

**Figure 5:**
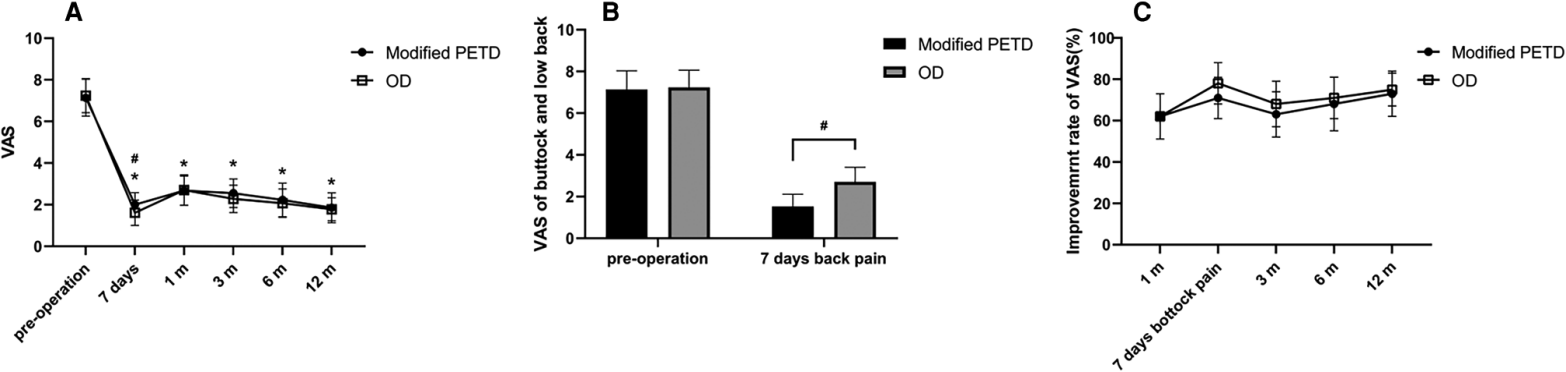
VAS score outcome values (**A**), VAS score outcome values for low back pain at 7 days postoperatively (**B**), and VAS score improvement rate (**C**). * indicates statistical significance compared within groups (*P* < 0.01), # indicates statistical significance compared between groups (*P* < 0.05).

**Figure 6:**
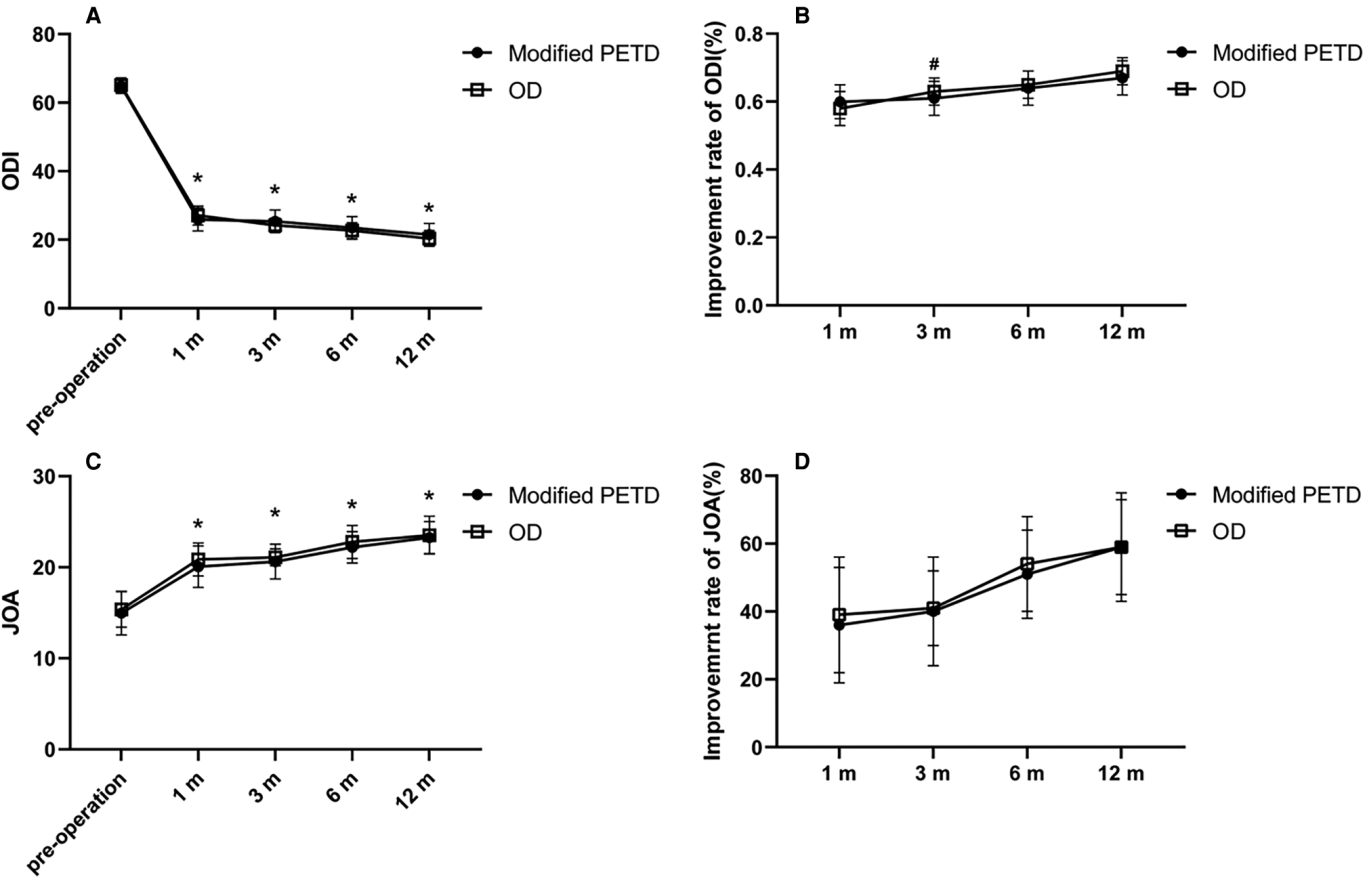
Outcome values (**A**) and improvement rates (**B**) for the ODI. Outcome values (**C**) and improvement rates (**D**) for the JOA. * indicates statistical significance compared within groups (*P* < 0.01). # indicates statistical significance compared between groups (*P* < 0.05).

All patients were interviewed by telephone at 1 month, 3 months, 6 months, and 12 months after the operation. The results showed that the VAS score outcome values decreased from 7.14 preoperatively to 2.00, 2.68, 2.55, 2.23, and 1.85 at 7 days, 1 month, 3 months, 6 months and 12 months postoperatively in the modified PETD group, with significant differences at each follow-up time compared with preoperatively (*P* < 0.01). Comparing between groups, the VAS score outcome value of 1.61 for gluteal pain at 7 days postoperatively in the OD group was better than that of 2.00 in the modified PETD group (*P* < 0.05) (Figure 5A), but the VAS score outcome value of 1.53 for low back pain (caused by surgical incision) at 7 days postoperatively in the modified PETD group was less severe compared to 2.70 in the OD group (*P* < 0.05) (Figure 5B). The improvement rates of VAS scores in the modified PETD group were 61.77%, 63.41%, 67.85%, and 73.47% at 1, 3, 6, and 12 months postoperatively, respectively. There was no significant difference compared to the OD group (*P* > 0.05) (Figure 5C). Within-group comparisons of the modified PETD group, the preoperative and postoperative outcome values for ODI (Figure 6A) and JOA (Figure 6C) showed dramatic improvements in both symptoms and function (*P* < 0.05). Compared with the OD group, the improvement rate of the ODI was statistically significant (*P* < 0.05) at 3 months postoperatively (Figure 6B), and there was no statistically significant (*P* > 0.05) improvement of the JOA during the follow-up period (Figure 6D).

**Figure 7:**
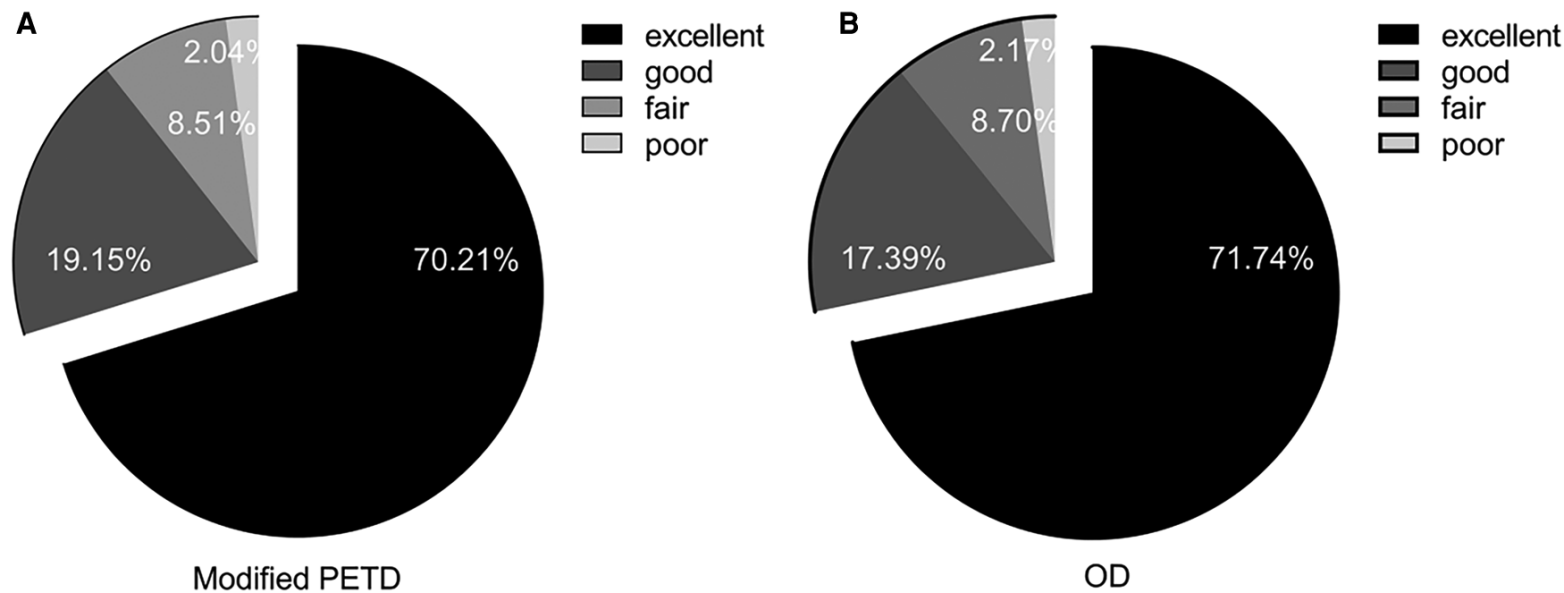
Modified MacNab scores for the modified PETD group (**A**) and for the OD group (**B**) at the postoperative follow-up at 12 months.

According to the modified MacNab criteria, 33 (70.21%) and 9 patients (19.15%) in the modified PETD group were considered “excellent” and “good” at 12 months postoperatively (Figure 7A), respectively; similarly, 33 (71.74%) and 8 patients (17.39%) in the OD group were considered “excellent” and “good,”, respectively (Figure 7B). Comparisons between groups were not statistically significant (*P* > 0.05).

### Recurrence and Complications

In the modified PETD group, two patients presented with recurrence at 15 days and 21 days postoperatively. We then treated them with OD, and the prognosis was favorable. Two patients in the OD group developed complications, a cerebrospinal fluid (CSF) leak and a hematoma. The former underwent intraoperative dural suturing and returned to the ward in a decubitus position for 12 h, where the headache caused by the CSF leak was relieved 5 days postoperatively. The latter presented with neurological compression due to a hematoma and recovered well after emergency debridement (Table 2).

**Table 2:** Recurrence and complications.

	No.	Age	Gender	BMI	Diagnosis	OP level	Recurrence days	Treatment
Modified PETD	1	47	Male	28.0	Recurrence	L4/5	11	OD
	2	45	Male	26.5	Recurrence	L4/5	16	OD
OD	1	72	Female	23.4	CSF leak	L4/5	–	Dural suture
	2	64	Female	25.2	Hematoma	L4/5	–	Debridement

*Different surgical approaches leading to recurrence and complications*.

*No., number CSF, cerebrospinal fluid*.

## Discussion

### Current Status of PETD Research

With the development of endoscopic techniques, surgeons have become more experienced, and patients prefer minimally invasive surgery, resulting in the rapid development of minimally invasive procedures for the spine (25). PETD has become the most used minimally invasive technique in recent decades due to its small incision, minimal damage to muscle and soft tissue structures, and minimal postoperative epidural fibrosis (20, 26).

It is generally accepted that PETD appears to be indicated for all types of LDH (27, 28). However, an RCT by Chen et al. (29) showed that PETD is more suitable for treating paracentral herniations, where a transforaminal approach facilitates visualization of the lesion. For median-type herniations, the limitations of the intervertebral foramen and dura lead to poorer clinical outcomes. This provides theoretical support for our study. All patients with gluteal pain had unilateral nerve root compression in the present study. Furthermore, the absence of iliac crest obstruction at the L4/5 level makes PETD a great advantage in the treatment of gluteal pain. However, PETD focuses on the surgical approach and removal of the nucleus pulposus and does not address or describe the annulus fibrous and posterior longitudinal ligament (20).

### Theoretical Basis for the Modification of the PETD

During clinical procedures, we found that stimulation of the patient’s fibrous annulus and posterior longitudinal ligament induced symptoms of gluteal pain. The patient showed considerable relief after denervation and removal of the fibrous annulus and posterior longitudinal ligament on the symptomatic side. Li et al. (30) showed that the sinus vertebral nerve (SVN) was divided into two types, the SVN deputy branch (type I) and the SVN main trunk (type II), with the SVN deputy branch entering the posterior lateral border of the disc and the SVN main trunk originating from the spinal ganglion and connecting to the sympathetic nerve via a traffic branch. Seventy (22.44%) SVN deputy branches and 23 (21.74%) SVN main trunks were found in the L4/5 intervertebral foramen. According to R et al. (31), part of the ascending branch of the SVN originates in the posterior longitudinal ligament, and microscopic observation of the sensory fibers of the posterior longitudinal ligament revealed that it receives a large number of traffic fibers of the SVN and forms a fiber network (32). When LDH is present, the production of inflammatory mediators leads to the transmission of inflammatory cytokines that hypersensitize SVN terminal receptors (33), which reduces the pain threshold and triggers buttock pain (34). Therefore, we hypothesized that gluteal pain might be associated with both and made improvements to the original.

### Modified PETD Has Great Potential to Treat Gluteal Pain Caused by L4/5 Disc Herniation

The modified PETD is safe and effective for treating gluteal pain caused by L4/5 disc herniation. The results showed that patients treated with the modified PETD showed a significant improvement postoperatively compared to preoperatively (*P* < 0.01). According to the modified MacNab score, 89.36% of patients were satisfied with the outcome 12 months after the procedure. There were no statistically significant differences in the VAS and JOA assessments of patients at 1, 3, 6, and 12 months after the modified PETD compared to those of the OD group (*P* > 0.05). The ODI was statistically significant only at 3 months postoperatively (*P* < 0.05), which we believe may be related to the subjective nature of the rating scale. In addition, there were no complications after treatment in the modified PETD group, although two patients experienced recurrence (4%), which we speculate may be related to the removal of the annulus fibrous tissue. Further studies are needed to determine whether the removal of the nucleus pulposus should be expanded. In summary, the modified PETD provides direct access to the lesion, relieves compression, and provides effective radiofrequency ablation of the SVN on the annulus fibrosus and posterior longitudinal ligament, relieving the patient’s symptoms. In addition, decompression of the lateral recess can also be accomplished with good results with modified PETD using a circular saw and a high-speed drill (35). The potential of the modified PETD for the treatment of LDH for gluteal pain was revealed.

### Comparison Between Modified PETD and OD

For patients with LDH with severe ossification or severe lumbar spinal stenosis, OD is an excellent treatment option. Complete extraction of the nucleus pulposus considerably reduces the possibility of recurrence. Furthermore, the adverse effects of recurrence should also be considered. A study by K et al. (36) showed that the reoperation rate of minimally invasive surgery patients was 3.1% higher than that of open surgery patients and that reoperation not only has negative psychological and physical impacts on the patients but also increases their financial burden. Nevertheless, due to the greater invasiveness, the patient has a longer recovery time and must endure the pain of a large incision (37), which can fail to heal in some diabetic patients. In addition, fixation of the nail bar system accelerates the degeneration of adjacent segments (38, 39). More importantly, when open surgery is performed, in addition to the removal of the lamina, the medial articular processes may be removed, and the surrounding ligament system and muscles may be destroyed. Extensive disruption of the posterior column may increase the risk of lumbar kyphosis (40) and lumbar spondylolisthesis (41). PETD avoids damage to the vertebral plates and spinous processes and greatly reduces the incidence of retroflection deformities (42). G et al. (43) showed no difference between PETD and OD for medium- to long-term pain and functional status. This is consistent with the results of our study. Similarly, this suggests that the two surgical strategies have the same efficacy. In addition, the modified PETD group had less postoperative low back pain (*P* < 0.01) and fewer complications than the OD group. For elderly patients with comorbidities, we should avoid the risks associated with general anesthesia and opt for safer local anesthesia (44). More importantly, for single-segment LDH, the modified PETD procedure appears to offer more benefit to patients than OD.

### Differential Diagnosis Related to Gluteal Pain

Buttock pain is often not a typical symptom of LDH. In clinical practice, it is often difficult for physicians to connect them, resulting in misdiagnosis and a delay in optimal treatment. Conditions that can cause buttock pain include deep gluteal syndrome and pain caused by the facet joint or sacroiliac joint (4, 45, 46). Deep gluteal syndromes are sciatica of nondiscogenic origin (47), including piriformis syndrome, gemelli-obturator internus syndrome, and ischiofemoral impingement syndrome (48). According to the literature by H et al. (49), the most common clinical feature of deep gluteal syndrome is pain in the buttocks, which is aggravated by prolonged sitting. In some patients, the straight leg raising test may be positive. These symptoms can be easily confused with the symptoms of buttock pain caused by LDH. Therefore, an accurate diagnosis of the disease before the operation is essential. In addition to a careful physical examination and empirical diagnosis, the surgeon should determine whether the patient’s symptoms are related to the lumbar spine by visualizing the MRI with the support of a radiological examination.

### Limitations

While this was a good retrospective study, there are still some limitations of which to be aware. A significant limitation is the retrospective nature of the study. The decision on surgical strategy was based on patient preferences. Second, the study population included only 93 patients from one hospital, which may have biased the results to some extent. Another is that this study reviewed patients with single-segment L4/5 disc herniations, and further research is still needed to determine the applicability of the modified PETD technique in patients with other segmental disc herniations. Third, further research is still required to explore the necessity of extended disc removal and preventing postoperative recurrence. Finally, when calculating the cost, we only counted the cost of minimally invasive surgery and not the cost of other procedures due to recurrence, which may lead to bias.

## Conclusions

The symptoms of gluteal pain due to L4/5 disc herniation should be highlighted in clinical practice. Modified PETD treatment is safe and effective and has the advantages of a lower complication rate, faster postoperative recovery, shorter length of stay, fewer anesthesia risks and lower cost of the procedure compared with OD. However, modified PETD has a higher recurrence rate, and reoperation caused by recurrence may increase the financial burden.

## Data Availability

The original contributions presented in the study are included in the article/Supplementary Material, further inquiries can be directed to the corresponding author/s.
